# Prenatal diagnosis of a rare form of congenital mid-ureteral stricture: a case report and literature revisited

**DOI:** 10.1186/1471-2490-7-8

**Published:** 2007-06-08

**Authors:** Milena Brugnara, Mariangela Cecchetto, Riccardo Manfredi, Michele Zuffante, Vassilios Fanos, Angelo Pietrobelli, Marco Zaffanello

**Affiliations:** 1 Department of Mother-Child and Biology-Genetics, University of Verona, Verona, Italy; 2 Department of Surgical-Gastroenterological Science, University of Verona, Verona, Italy; 3 Department of Morphological-Biomedical Science, University of Verona, Verona, Italy; 4 Department of Nuclear Medicine, University of Verona, Verona, Italy; 5 Department of Pediatrics and Clinical Medicine-Section of Neonatal Intensive Care Unit, University of Cagliari, Cagliari, Italy

## Abstract

**Background:**

Congenital mid-ureteral stricture is a rare malformation of the ureter leading to prenatal and neonatal hydronephrosis. Site characterization of the narrowing is important to optimize the surgical approach to the newborn affected by hydronephrosis.

**Case presentation:**

We report a female EM with a rare form of hydronephrosis, (i.e. mid-ureteral stricture) which was detected early during pregnancy by imaging techniques. During fetal life both conventional fetal Ultrasound and maternal Magnetic Resonance Imaging (MRI) were used to diagnose the obstruction. Magnetic Resonance pyelography and retrograde Ureteropyelography were performed after delivery and before surgical correction and confirmed the finding.

Furthermore, we revisited the literature using online MEDLINE and EMBASE databases. The literature reported only a few cases of prenatal diagnosis of early onset mid-ureteral stricture.

**Conclusion:**

Mid-ureteral stricture is a rare cause of prenatal hydronephrosis. The diagnosis should not be delayed in order to apply the appropriate surgical approach. As a result, we showed the usefulness of fetal MRI and postnatal Magnetic Resonance pyelography, in the event that radionuclide renography with Tc-MAG3 was less informative, to allow the detection of the site of ureteral narrowing. Intrasurgical retrograde ureteropyelography confirmed these findings.

## Background

Screening pregnancies with ultrasonography (US) may aid characterization of hydronephrosis in the uterus [1]. The hydronephrosis could be mono-lateral or bilateral. Furthermore, the stricture may compass from the ureteral-pelvic junction, through different levels of the ureter, to the ureteral-bladder junction. Site characterization of the narrowing is important because it could suggest the best surgical approach, and, in particular, the urgency of surgical approaches to newborns affected with hydronephrosis [2].

Incidence of genital-urinary anomalies ranges from 2 to 9 every 1,000 pregnancies, with a male/female ratio of 2:1 [3]. Prenatal US detects several pathological conditions of the urinary tract, (i.e., multicystic dysplastic kidney disease, polycystic kidney disease, agenesis or dysplasia of the kidney) and other rare pathological conditions. Hydronephrosis is the most common of these diseases [4]. In the majority of cases (63 %) prenatal hydronephrosis can be associated with normal renal physiology. Frequently this finding could spontaneously return to normal during the first year of life [5], requiring only a clinical follow-up. However in the minority of cases hydronephrosis is a severe pathologic condition that requires a surgical approach. The most frequent causes of hydronephrosis that require surgical correction are ureter-pelvic junction obstruction (11 %), vesicoureteral reflux (9 %) and vesicoureteral junction obstruction (4 %) [6]. In line with these findings, we report a case of a rare prenatal form of hydronephrosis describing the diagnostic procedures with imaging techniques performed before the surgical approach.

## Case presentation

**Figure 1:**
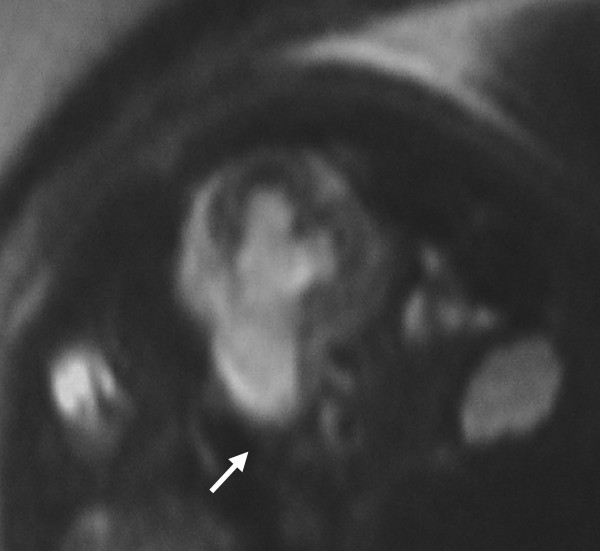
Fetal Magnetic Resonance Imaging (MRI) imaging: T2-weighted sequences. Axial Half Fourier Rapid Acquisition with Relaxation Enhancement (RARE) T2-weighted images shows dilation of the middle third of the ureter and coexist homolateral upstream hydronephrosis.

A female newborn EM was full term born after normal pregnancy and delivery. However, during the pregnancy she demonstrated a progressive worsening of hydronephrosis of the left kidney at routine screening US. In particular, at 21 weeks of gestational age the subject showed a pelvis dilatation (7 mm) of the left kidney. However, hydronephrosis severely worsened (12.5 mm) 5 weeks before the delivery. Furthermore, routine US suspected a dilatation of left proximal ureter and a reduction of the cortical thickness of the left kidney. For this reason, fetal MRI was allowed to investigate this finding. The fetal MRI suspected a left ureteral stricture since she showed a dilatation of the middle third of the ureter. In addition, a homolateral upstream hydronephrosis was shown in the subject (Figure 1).

The US performed after birth (at 5 days of life) showed a worsening of the left pelvis dilatation (20 mm), whereas the right pelvis was normal. Routine blood and urine bacterial analyses were normal. The Glomerular Filtration Rate (GFR) calculated by the Schwartz formula was normal (64.6 ml/min/1.73 m^2^).

A voiding cystourethrogram was performed on the subject at 1 month of age, showing no vesicoureteral reflux, however the follow-up US confirmed a worsening of the hydronephrosis.

At 50 days of life, a diuretic radionuclide renography was performed on the subject with Technetium-99m mercaptoacetyltriglycine (Tc-MAG3). This technique showed the left kidney was seriously enlarged and hypo-functioning (20 % of total renal function). In addition, a mild dilatation of the intra-renal excretion pathways was shown with a corticalized calyceal system. Furthermore, the evaluation of roengtenographic urine outflow was very complicated because there was a low kidney function and very low urine production. For this reason, the renography with Tc-MAG3, after only a mild left intensification, showed a drop pattern without variation during the diuretic phase induced by intravenous diuretics (furosemide).

**Figure 2:**
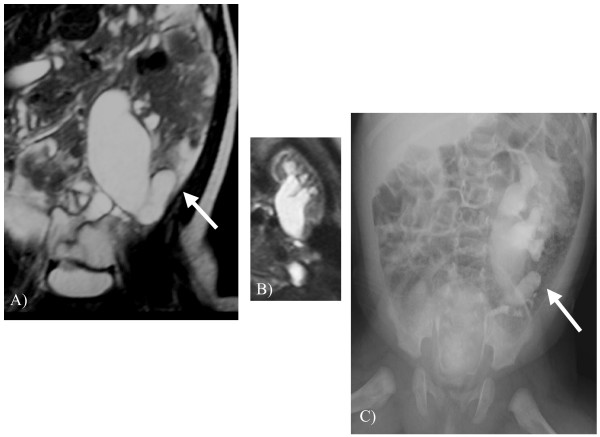
Magnetic Resonance Imaging (MRI) coronal T2-weighted images (Panel A), coronal MR urography (Panel B), and intrasurgical retrograde ureteropyelogram (Panel C). On coronal T2-weighted images and coronal MRI urography, can be depicted an obstruction of the middle third of the ureter, with upstream hydro-urethronephrosis, that was subsequently confirmed by intrasurgical retrograde ureteropyelogram (Panel C).

The subject was investigated at 2 months of age by MR urography (Figure 2, Panels A and B). The examination showed a significant worsening of the left pelvis dilatation (anterior-posterior diameter of 30 mm) with a severe extrarenal enlargement. In addition, the test showed the proximal ureter was dilated by almost 2.8 cm and then stopped and flexed. The intrasurgical retrograde ureteropyelogram that was subsequently performed confirmed a mid-ureteral stenosis (Panel C).

Surgical resection-anastomoses of the obstructed left ureteral tract allowed establishing normal ability of urine outflow [7].

The histological analysis of the resected ureteral tract (2.8 cm of length) showed an increase of collagen and fibrosis in both *lamina propria *and *tunica muscularis*.

Hydronephrosis is the most frequent abnormality of the fetal urinary tract detected by US. Simply it means a dilatation of the renal pelvis. The dilatation rate allowed a categorization of the hydronephrosis severity. The renal pelvis with anterior-posterior (AP) diameter more than 7 mm prior to 20 weeks of gestational age and 9–15 mm after 30 weeks of gestational age should be considered as moderate hydronephrosis [4,8]. Moreover, a pelvis AP diameter greater than 7 mm at the third trimester of pregnancy would always need a close follow-up [1].

There are many etiologies involved in obstructive uropathies. Rare causes of hydronephrosis are congenital mid-ureteral strictures. The ureteral strictures are narrowing of the lumen of the ureter [9]. Strictures are the decrease of ureteral lumen diameter by 60%. These obstructions are usually due to valves [10]. The ureteral valves are transverse folds of ureteral mucous involved in the strictures. Besides, these strictures can be due to anomalous organization of the ureteral musculature, which may be caused by alterations in the fetal canalization process or consequently to insufficient vascular supply. These ureteral tracts have a reduction of smooth muscle cells. In severe strictures, the smooth muscle layer should be replaced by fibrous tissue and associated with other abnormalities of the urinary tract [11-13].

Unfortunately, obstructive uropathies may result in both tubular damage and decreased number of nephrons already during fetal life [14]. For the appropriate management, it could be interesting to obtain the exact localization of the ureteral tract obstruction as soon as possible [15]. Therefore, US performed during pregnancy are not usually able to characterize the exact cause of hydronephrosis. In our subject, fetal MRI was able to detect/suspect a ureteral obstruction. However, only postnatal MRI pyelography of the urinary tract confirmed the obstruction at ureteral level. This case of both severe ureteral obstruction and reduced kidney function did not show a very informative radionuclide renography by Tc-MAG3. Notwithstanding the successful pyeloplasty, only minimum function recovery can be expected in kidneys with poor function and hydronephrosis diagnosed during pregnancy [9]. This may be because a chronic renal failure frequently occurs if the neonate at birth has got significant kidney damage from chronic prenatal obstruction [14]. Finally imaging techniques, in particular MRI pyelography, could rapidly and accurately describe the morphological features of a dilated urinary tract with information as well as to the level of obstruction [16].

## Conclusion

To our knowledge, the mid-ureteral tract is rarely observed as the prenatal onset of urinary tract obstruction [9,17,18]. Recently Smith *et al *looked at 4 new cases of mid-ureteral strictures and reviewed 13 previously reported cases. These authors found that the retrograde pyelography was diagnostic in all 17 cases [12]. Furthermore, Hwang et al reported 5 cases of this rare abnormality presented as prenatal hydronephrosis [9]. In these cases, both renal ultrasound and radionuclide renography did not reliably demonstrate the site of obstruction. Therefore, a retrograde pyelography performed at the time of surgical correction improved the identification of the site of obstruction [9]. Finally, to investigate the congenital mid-ureteral stricture we showed the feasibility of fetal MR and then postnatal MR pyelography, in the case that radionuclide renography with Tc-MAG3 was less informative, to allow detecting the site of narrowing. Intrasurgical retrograde ureteropyelography confirmed these findings.

## Abbreviations

US = ultrasonography; MRI = magnetic resonance imaging; Tc-MAG3 = technetium-99m mercaptoacetyltriglycine.

## Competing interests

The author(s) declare that they have no competing interests.

## Authors' contributions

MB contributed to this work in collecting and analyzing the data; MC, RM and MZU contributed to this work in analyzing the data; VF was involved in critically revising the manuscript; MZA contributed to the conception, analysis and interpretation of the results.

## Pre-publication history

The pre-publication history for this paper can be accessed here:


